# Detection of xerostomia, Sicca, and Sjogren’s syndromes in a national sample of adults

**DOI:** 10.1186/s12903-021-01917-1

**Published:** 2021-10-27

**Authors:** Indre Stankeviciene, Alina Puriene, Diana Mieliauskaite, Lina Stangvaltaite-Mouhat, Jolanta Aleksejuniene

**Affiliations:** 1 Institute of Dentistry, Faculty of Medicine, Vilnius University, Vilnius, Lithuania; 2 Department of Experimental, Preventive and Clinical Medicine, State Research Institute Centre for Innovative Medicine, Vilnius, Lithuania; 3 Oral Health Centre of Expertise in Eastern Norway, Oslo, Norway; 4 Department of Clinical Dentistry, Faculty of Health Sciences, The UiT Arctic University of Norway, Tromsø, Norway; 5 Department of Oral Health Sciences, Faculty of Dentistry, University of British Columbia, Vancouver, Canada

**Keywords:** Xerostomia, Sicca syndrome, Sjogren's syndrome, Saliva, Adults

## Abstract

**Objectives:**

To assess the prevalence and determinants of xerostomia among adults and identify how many of the ones experiencing xerostomia have Sicca and Sjogren's syndromes.

**Materials and methods:**

This cross-sectional study included 1405 35–74-year-old Lithuanians (51.7% response rate) from the five largest Lithuanian cities and 10 peri-urban and rural areas that were randomly selected from each of the 10 Lithuanian counties. Xerostomia was determined by the self-reported experience of dry mouth as "often" or "always". A dentist diagnosed Sicca syndrome by unstimulated whole sialometry and the Schirmer's test, and all cases were referred to a rheumatologist to confirm Sjogren's syndrome. Self-reported questionnaires collected data about the determinants.

**Results:**

The prevalence of xerostomia was 8.0% (n = 112), Sicca syndrome was diagnosed for 8 participants (0.60%), and Sjogren's syndrome for 2 participants (0.14%), with this being the first time it was diagnosed. Experiencing xerostomia was associated with older age (OR 1.7, 95% CI 1.1–2.6), urban residence (OR 3.3, 95% CI 1.6–5.0), presence of systemic diseases (OR 2.5, 95% CI 1.4–3.3), and the use of alcohol (OR 0.6, 95% CI 0.4–0.9). The higher proportion of participants with Sicca syndrome involved females, of older age, having systemic diseases, and using medications.

**Conclusions:**

The prevalence of xerostomia was 8.0% and the determinants of xerostomia were older age, urban residence, systemic diseases, and absence of using alcohol. In total, 0.6% of participants had Sicca syndrome, which was more prevalent among females, older subjects, those with systematic diseases, and those using medications. Sjogren's syndrome was diagnosed in 0.14% of participants*.*

*Clinical relevance*

Dental clinicians need to be trained to identify potential Sjogren's syndrome cases.

## Introduction

Aging subjects increasingly suffer from dry mouth conditions; therefore, in many industrialized countries, with longer life expectancies, dry mouth is becoming an important consideration that dental professionals should be aware of [1]. The term "dry mouth" usually covers one of two conditions: xerostomia or hyposalivation [2]. Xerostomia, the most prevalent form of mouth dryness, is defined as a subjective sensation that is usually assessed directly by asking individuals about their dry mouth experience [3]. Hyposalivation is objectively diagnosed and based on the amount of saliva produced [4]. Mouth dryness has been associated with poor oral health, as indicated by higher rates of dental caries, periodontal diseases, and oral infections; prosthetic problems were also observed in patients with dry mouth [3]. Dry mouth can also impact a person's general health, nutrition, and quality of life; it may also be the first sign of various chronic diseases [5].

In comparison, Sicca syndrome is diagnosed when a person suffering from a dry mouth also experiences a lack of tear flow or another xerosis. The etiology of such a condition may be autoimmune or non-autoimmune (medications, radiotherapy, systemic diseases). Patients suffering from Sicca syndrome have an increased risk for Sjogren’s syndrome (based on Sjogren’s classification criteria) or Sjogren's syndrome associated to other autoimmune diseases was confirmed (based on existing Sjogren’s syndrome symptoms together with other systemic diseases of connective tissues). Mouth dryness may be among the most apparent early symptoms, allowing us to suspect Sjogren's syndrome. Therefore, dental clinicians might be the frontline healthcare providers to detect dry mouth and its corresponding conditions. Sjogren's syndrome can be defined as an autoimmune disease of multifactorial etiology resulting in hypofunction of both the salivary and tear glands, later impacting several organ systems. It has also been associated with 16 times greater risk for non-Hodgkin’s B-cell lymphoma [6, 7]. Under-diagnosis of Sjogren's syndrome is common, mainly due to multiple criteria needed for its diagnosis and lack of relevant expertise among healthcare specialists [6, 7]. The average diagnosis time for Sjogren's syndrome is nearly 6 years. It is important to note that the timely identification of this disease would allow its early treatment and prevention of complications [7].

The current study assessed the prevalence of xerostomia in the Lithuanian adult population and examined how many of those with xerostomia also have Sicca and Sjogren's syndromes. The study tested several potential sociodemographic and general health-related determinants of xerostomia and Sicca syndrome.

## Materials and methods

The current study was performed in a dental clinical setting as part of the cross-sectional national oral health survey. The data were collected from 2017 to 2019. The cluster random sample included 35- to 74-year-old subjects from the five largest Lithuanian cities and 10 peri-urban and rural areas that were randomly selected from each of the 10 Lithuanian counties. In each pre-selected location, the selected number of participants was extracted from the patient lists at primary health care institutions, subsequently invited to participate in the study. Subjects who were in military service, prison, or special care institutions, along with those who were mentally disabled or not present in the country at the time of data collection, were excluded.

Calculations for the necessary sample size showed that we needed a minimum of 300 participants from each pre-selected age group: 35–44, 45–54, 55–64, and 65–74. The calculated sample size was multiplied by 1.5 to adjust for the study design and further increased due to an expected 50% non-participation rate [8]. As such, a total of 2716 adults were invited to participate, of which 1405 agreed (response rate 51.7%).

The survey included questions from three validated questionnaires, namely the WHO Oral Health Questionnaire for Adults, the Perceived Stress Scale (PSS-10), and the Sjogren's Questionnaire [9–11]. In addition, we included question “*how often does your mouth feel dry?"* [4] and collected information about body mass index, systemic diseases, and the use of medications.

Two persons independently translated the survey questionnaire back and forth between English and Lithuanian, then between Russian and Polish. Subsequently, inconsistencies were discussed and corrected. The survey questionnaire was pre-tested in a group of 10 subjects who did not participate in the main survey.

**Fig. 1:**
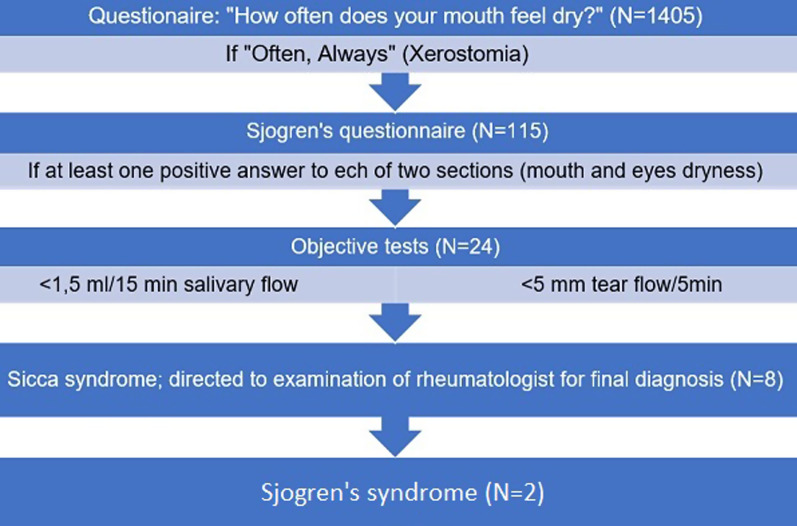
Flow chart of the selection of the participants with Sicca syndrome

The study outcomes were the presence of xerostomia, Sicca, and Sjogren's syndromes. Figure 1 presents a flow chart of how the subjects with dry mouth conditions were identified. Firstly, all study participants were asked a question "*how often does your mouth feel dry?"* (responses: never, sometimes, often, always) [4]. Based on previous studies, the xerostomia group was comprised of those who indicated feeling mouth dryness "often" or "always"; therefore, others served as a comparison group. Furthermore, those who reported xerostomia completed an additional Sjogren's questionnaire (Fig. 1). For those participants who reported eye dryness in addition to mouth dryness, the following objective tests were performed: the whole unstimulated sialometry and the Schirmer's test. Before the examination the participants were asked not to eat or brush teeth for two hours, then participants were asked to spit out all accumulated saliva into gradated tube; hyposalivation was considered when the amount of saliva being less than 1.5 ml in 15 min. Schirmer’s tests were used to measure the tearflow: two strips were used for the left and right eyes, placing them in approximately center of lower lid, and tear flow < 5 mm in 5 min was considered as pathology. After unstimulated whole sialometry and Schirmer’s tests, eight participants were diagnosed with Sicca syndrome. The subjects from the Sicca syndrome group were referred to a rheumatologist to confirm the Sjogren’s syndrome diagnosis. The Sjogren’s syndrome was diagnosed according to the 2016 ACR/EULAR criteria based on positive pathohistology (focal sialadenitis), and positive antibodies (anti-SSA/Ro).

The operationalization of the study variables is presented in Table 1. The following potential determinants of xerostomia were tested: socio-demographic characteristics (age, gender, residence, education) and general health status (systemic diseases, medications, alcohol use, smoking, perceived stress, body mass index).

**Table 1:** Operationalization of the study variables and their categorization for analyses

Variables	O Original measurement ( (codes)	Bivariate analyses (codes)	Multivariate analyses (codes)
**Study Outcomes:** Xerostomia	Self-reported mouth dryness: '1' never, '2' sometimes, '3' often, '4' always	'1' never/sometimes, '2' often/always	'1' control group, '2' xerostomia group
Sicca syndrome	'1' Sicca syndrome '2' Non-Sicca syndrome	'1' Objective symptoms of Sicca syndrome '2' No objective symptoms of Sicca syndrome	
**Indicators:**			
Gender	1) males, 2) females	'1' males, '2' females	'1' males, '2' females
Age	In full years	'1′ 35–46 years, '2′ 47–60 yrs.; '3′ 61–74 yrs	'1′ 35–60 yrs.; '2′ 61–74 yrs
Residence	Location of residence: '1' urban; '2' peri-urban; '3' rural	‘1’ urban; ‘2’ peri-urban; ‘3’ rural	'1' urban; '2' peri-urban/rural
Education	In full years	'1' ≤ secondary; '2' college; '3' university +	'1' secondary or less; '2' college/university or more
Systemic diseases (Total number)	Any systemic condition	'1' none; '2' one, '3' two or more conditions	'1' none; '2' one or more
Medications (Total number)	Any medication	'1' none; '2' one, '3' two or more	'1' none; '2' one or more
Perceived stress Total stress score (%)	10 questions (responses: '0' never; '1' rarely; '2' sometimes; '3' often; '4' very often. calculated from responses 3 and 4 and adjusted for the number of answers	'1' low; '2' medium, '3' high/very high	'1' low; '2' medium/high/very high
Body Mass Index (BMI)	Based on weight & height (BMI = kg/m^2^)	'1' BMI ≤ 25.0; '2' BMI 25.1–29.9; '3' BMI ≥ 30	'1' underweight/normal; '2' overweight/obese
Smoking	'1' no; '2' yes	'1' no; '2' yes	'1' no '2' yes;
Daily alcohol use in units^1^	Alcohol consumed in the past month: '0' none; '1' one unit; '2' two units, '3' units; '4' units; '5' five or more	'1' no; '2' yes	'1' no; '2' yes

^1^One unit of alcohol: one beer or glass of wine or a small goblet of heavy liquor

SPSS Version 25.0 software was used for statistical analyses. For the comparison of proportions (bivariate analyses) between the two study groups (xerostomia and comparative), the Chi-Square or Fisher's Exact tests (when conditions for the Chi-Square test were not met) were employed. The multivariable binary logistic regression analysis tested the joint effect of potential determinants (Table 1) in regard to the presence of xerostomia (study outcome). Model 1 tested a total of four socio-demographic predictors while Model 2 tested three socio-demographic (education had to be excluded from Model 2 due to a multicollinearity problem) and six general health-related determinants. For both bivariate and multivariable analyses, the threshold for significance was set at *p* < 0.050.

## Results

The present study included 466 males and 939 females. The prevalence of xerostomia was 8.0% (n = 112), and Sicca syndrome was diagnosed in 0.6% (n = 8) of participants. The higher proportion of participants who experienced xerostomia were female, older subjects, having lower education, using medications, having systemic diseases, and higher levels of perceived stress (Table 2). Similarly, in the Sicca syndrome group there were more females, older subjects, and those having systemic diseases and using medications (Table 2). In the multivariable model, four predictors were significantly associated with xerostomia: older age (OR 1.7, 95% CI 1.1–2.6), urban residence (OR 3.3, 95% CI 1.6–5.0), presence of systemic diseases (OR 2.5, 95% CI 1.4–3.3), and using alcohol (OR 0.6, 95% CI 0.4–0.9) (Table 3).

**Table 2:** Distribution of predictors-comparisons among the control versus xerostomia, and control versus Sicca syndrome groups

Predictors	Study groups					
	Control N (%)	Xerostomia N (%)	Significance^1^	Control N (%)	Sicca syndrome N (%)	Significance^1^
**Gender**	1293	112		1293	8	
Males	440 (34.0)	26 (23.2)	0.020	440 (34.0)	0 (0.0)	0.043
Females	853 (66.0)	86 (76.8)		853 (66.0)	8 (100.0)	
**Age groups**	1293	112		1293	8	
Younger (35–46 years)	387 (29.9)	12 (10.7)	< 0.001	387 (29.9)	0 (0.0)	0.007
Middle (47–60 years)	460 (35.6)	33 (29.5)		460 (35.6)	1 (12.5)	
Older (61–74 years)	446 (34.5)	67 (59.8)		446 (34.5)	7 (87.5)	
**Residence**	1293	112		1293	8	
Urban	917 (70.9)	89 (79.5)	0.093	917 (70.9)	8 (100.0)	0.355
Peri-urban	227 (17.6)	11 (9.8)		227 (17.6)	0 (0.0)	
Rural	149 (11.5)	12 (10.7)		149 (11.5)	0 (0.0)	
**Education**	1293	112		1293	8	
Secondary or less	500 (38.7)	57 (50.9)	0.040	500 (38.7)	4 (50.0)	0.903
College	336 (26.0)	23 (20.5)		336 (26.0)	2 (25.0)	
University and higher	457 (35.3)	32 (28.6)		457 (35.3)	2 (25.0)	
**Systemic diseases**	1293	112		1293	8	
None	638 (49.3)	22 (19.6)	< 0.001	638 (49.3)	0 (0.0)	0.006
One	521 (40.3)	58 (51.8)		521 (40.3)	7 (87.5)	
Two+	134 (10.4)	32 (28.6)		134 (10.4)	1 (12.5)	
**Use of medications** 1293		112		1293	8	
None	574 (44.4)	14 (12.5)	< 0.001	574 (44.4)	0 (0.0)	0.022
One	586 (45.3)	67 (59.8)		586 (45.3)	7 (87.5)	
Two+	133 (10.3)	31 (27.7)		133 (10.3)	1 (12.5)	
**Body Mass Index** 1293		112		1293	8	
≤ 25.0	471 (36.4)	31 (27.7)	0.057	471 (36.4)	3 (37.5)	0.552
25.1–29.9	434 (33.6)	36 (32.1)		434 (33.6)	4 (50.0)	
≥ 30.0	388 (30.0)	45 (40.2)		388 (30.0)	1 (12.5)	
**Smoking**	1293	112		1293	8	
No	981 (75.9)	87 (77.7)	0.667	981 (75.9)	7 (87.5)	0.688
Yes	312 (24.1)	25 (22.3)		312 (24.1)	1 (12.5)	
**Alcohol use**	1293	112		1293	8	
No	510 (39.4)	50 (44.6)	0.281	510 (39.4)	5 (62.5)	0.277
Yes	783 (60.6)	62 (55.4)		783 (60.6)	3 (37.5)	
**Self-perceived stress** 1130 95				1130	8	
Low	367 (32.5)	16 (16.8)	< 0.001	367 (32.5)	1 (12.5)	0.511
Medium	695 (61.5)	67 (70.5)		695 (61.5)	4 (50.0)	
High	68 (6.0)	12 (12.6)		68 (6.0)	3 (37.5)	

^1^ Chi-square or Fischer's Exact test when requirements were not met

**Table 3:** Predictors for xerostomia in Lithuanian adults (binary multivariable logistic regression analyses)^1^

Predictors	OUTCOME: Presence of xerostomia No '0' (N = 112), Yes '1' (N = 1291)					
	Model 1 Nagelkarke R^2^ = 0.756 Significance: *p* < 0.001			Model 2^1^ Nagelkarke R^2^ = 0.761 Significance: *p* < 0.001		
	Odds Ratio	95% CI	Tolerance	Odds Ratio	95% CI	Tolerance
**Socio-demographic factors**						
Gender	1.1	0.7; 1.6	0.976	1.2	0.8; 1.9	0.878
Age	1.6	1.2; 2.3	0.964	1.7	1.1; 2.6	0.789
Residence	0.3	0.2; 0.4	0.952	3.3	1.6; 5.0	0.934
Education	0.4	0.3; 0.5	0.924			
**Status of general health**						
Systemic diseases				2.5	1.4; 3.3	0.923
Medication use				1.4	0.9; 2.2	0.792
Stress				1.2	0.7; 2.0	0.943
Body Mass Index				0.9	0.6; 1.4	0.903
Smoking				0.7	0.4; 1.2	0.917
Alcohol use				0.6	0.4; 0.9	0.902

^1^ Education was excluded from Model 2 due to the multicollinearity problem

Primary Sjogren's syndrome was diagnosed in two of eight participants (0.14%) having Sicca syndrome, and this was their first-time diagnosis. For the two other participants, Sjogren's syndrome associated to other autoimmune diseases was confirmed, while two subjects were diagnosed with non-autoimmune Sicca syndrome, and the last two did not receive the final diagnosis (Fig. 1).

## Discussion

In the present study, the prevalence of xerostomia was 8.0%, (n = 112), 0.6% (n = 8) for Sicca syndrome, and 0.14% (n = 2) for Sjogren’s syndrome. Awareness of common determinants of xerostomia and Sicca syndrome, effective identification of xerostomia, and knowledge about what steps should be taken in order to confirm Sicca and Sjogren’s syndromes are essential when working with ageing populations. The current study demonstrated that the detection of dry mouth conditions and the diagnosis of Sjogren's syndrome may be part of dental practices if dentists are trained. Although knowledge held by dental clinicians was not examined in this study, it is likely that lack of this specific knowledge may lead to underdiagnosis of dry mouth conditions and Sjogren’s syndrome. The strength of the present study is that it included a representative national sample of adults, of which three study groups were formed: (1) subjects with xerostomia, (2) Sicca syndrome subjects who are known to be at increased risk to have Sjogren's syndrome, and (3) the comparative group (without xerostomia and Sicca syndrome). The multivariable regression, including multiple determinants, explained a substantial proportion of the variation in xerostomia [12].

In our study, the Sicca group was relatively small, thus the findings should be interpreted with caution. Other limitations of the present study are the inherent shortcomings of a cross-sectional study design that does not allow causal inferences, e.g., interpreting significant determinants of xerostomia as its potential causes. Potential information bias due to self-reporting of either xerostomia or its predictors was also possible [13]. Another limitation was that objective tests, due to time restriction in clinical settings, were used only for participants who reported xerostomia, and the real prevalence of Sicca syndrome could not be established. Future similar studies should consider including both subjective and objective measurements for all participants. It should be noted that in our study 67% of participants were females, but such proportions of females correspond to the national 2018 statistics data, as there were more females (53.8%) than males, in addition that in the age group 65+ the proportion of females was even higher (66.3%) [14]. Besides, at the time of the survey, some males were undergoing military service, or had employments outside Lithuania.

Our study found that the prevalence of xerostomia in Lithuanian adults was 8.0%, which is at the lower end compared to global reports (ranging from 1.0 to 80.0%) [15, 16]. The wide variation in global prevalence rates of xerostomia may be due to the measurements chosen or sampling-related variations (differences in age, gender, health) [17]. In comparison to other national studies, the prevalence of xerostomia in Lithuanian adults is similar to the 10.0% prevalence reported in a New Zealand study, which included adults and elderly. It was also similar to a Brazilian study (11.0% in 59-year-olds) where comparable measurements were used, namely xerostomia was diagnosed if participants answered: "often" or "always" to the question "*How often do you feel oral dryness?*" [19]. It is not clear whether we should focus on those frequently experiencing xerostomia known to be at increased risk for oral health conditions and Sjogren’s syndrome only, or we should examine all adults to identify different levels of xerostomia.

Similar to studies performed in New Zealand and Sweden, we found that older age was related to xerostomia [19, 20]. According to two reviews, one of the reasons for age-related xerostomia is polypharmacy, which is more common in older individuals [21, 22]. However, this was not supported by the findings of our multivariable analysis. This finding was somewhat unexpected, as it is known that more than 500 types of medications can cause xerostomia [22]. We think that the disappearance of the significant effect of medication use observed in the multivariable analysis might be due to the control of other determinants such as the presence of systemic diseases, stress, body mass index, smoking, and alcohol use. A significant association was found between the presence of xerostomia and self-reported systemic diseases. The self-reported diseases specified by our participants were hypertension, diabetes mellitus, thyroid dysfunction, gastroesophageal reflux disease, osteoporosis, and others. According to the literature, xerostomia is often associated with the above-mentioned systemic conditions [21, 23]. We found that there was an association between urban residence and higher odds of xerostomia. We do not have definitive explanations for these findings, but we think this may be due to less healthy lifestyle being more common in urban than in rural/peri-urban residents. In addition, we found a significant relation between the use of alcohol and lower odds of xerostomia. Our findings are in accordance to the two previous studies demonstrating that higher proportion of participants who did not use alcohol reported xerostomia more often [24, 25]. In order to acquire a more clear insight, future studies should measure the amount of alcohol use and associate it with xerostomia [24].

Self-perceived stress was significantly associated with xerostomia in bivariate, but not multivariable, analyses, i.e., when controlled for other determinants such as sex, age, residence, systemic diseases, body mass index, smoking, alcohol consumption, and use of medications. In comparison, a few previous studies associated stress with xerostomia but not with hyposalivation [26, 27]. The researchers suggested that acute and chronic stress may impact salivation differently, and that there might be differences related to personality characteristics and sex [26, 27]. Females, compared to males, respond to stressors differently, probably due to sex-related variations in hormones such as ACTH, cortisol, and DHEA as part of the HPA axis [28]. Future studies should also explore how stress is related to dry mouth and Sjogren’s syndrome.

In our study, the prevalence of the objectively validated Sicca syndrome was 0.6%. This prevalence was lower than that observed in Salisbury, UK, where 4.4% of adults had both eye and mouth dryness [29]. It should be mentioned that the sample in the UK study included sex-related findings, namely that Sicca syndrome is more common in females than in males which is similar to the aforementioned study. Sicca syndrome is often associated with autoimmune processes and about 80% of patients diagnosed with autoimmune diseases are females [30]. The Salisbury study demonstrated a dose–response relationship between Sicca syndrome and the use of medications, while we found a relationship between Sicca and the use of medications in the bivariate analysis only. In contrast to the Schein et al*.* study, the majority of our Sicca group participants reported only one medication and one systemic disease [29]. In the future, larger studies are needed to identify potential determinants of Sicca syndrome.

It was challenging to compare our results with other studies focusing on Sicca syndrome, as there is confusion in the literature stating that the presence of mouth and eye dryness indicates Sjogren's syndrome. Importantly, suspected Sjogren’s syndrome needs to be validated by other objective criteria including the presence of specific autoantibodies. Our study results may illustrate the existing difference between Sicca and Sjogren’s syndrome. The 0.14% prevalence of Sjogren’s syndrome found in our study is in line with global rates ranging from 0.01 to 3.0% [31]. Our findings suggest that dentists can uncover suspected Sjogren’s syndrome cases, given they perform a thorough clinical examination. Dental practitioners need to acquire knowledge about mouth dryness and its determinants, as well as learn how to identify xerostomia. We propose training dental practitioners in diagnosing xerostomia in high-risk patients, then practitioners should be trained in follow-up diagnostic methods that would allow them to differentiate between the suspected Sjogren’s syndrome and the need for referral to a rheumatologist. This should be practiced in all dental offices; consequently, this might be a first step towards detecting both Sicca and Sjogren’s syndromes. In our study, the majority of participants in the Sicca syndrome group had the autoimmune or non-autoimmune condition (others did not get a final diagnosis). For half of the Sicca syndrome group participants, it was the first time this condition was diagnosed. In the present study, two out of eight Sicca syndrome group participants explained that they experienced eye and mouth dryness and sought help from their general health practitioners; however, these general health practitioners did not refer them to rheumatologists. This might indicate that general practitioners may not have sufficient knowledge in detecting and or managing mouth dryness. Therefore, training about mouth dryness and other related conditions is required for dentists and other health practitioners.

## Conclusions

The prevalence of xerostomia was 8.0% and related to older age, urban residence, and systemic diseases. The prevalence of Sicca syndrome was 0.6%, which was more prevalent among females, in older age groups, and in those having systematic diseases and using medications. The prevalence of Sjogren's syndrome was 0.14%. Our findings support the notion that dental clinicians, if trained, can contribute towards the timely detection of Sjogren’s syndrome.

## Data Availability

The dataset supporting the conclusions of this article is available from the corresponding author upon reasonable request.
